# Recurrent Pregnancy Loss Etiology, Risk Factors, Diagnosis, and Management. Fresh Look into a Full Box

**DOI:** 10.3390/jcm12124074

**Published:** 2023-06-15

**Authors:** Akbayan Turesheva, Gulzhanat Aimagambetova, Talshyn Ukybassova, Aizada Marat, Perizat Kanabekova, Lyazzat Kaldygulova, Ainur Amanzholkyzy, Svetlana Ryzhkova, Anastassiya Nogay, Zaituna Khamidullina, Aktoty Ilmaliyeva, Wassim Y. Almawi, Kuralay Atageldiyeva

**Affiliations:** 1 Department of Normal Physiology, West-Kazakhstan Marat Ospanov Medical University, Aktobe 030000, Kazakhstana.ainur.82@mail.ru (A.A.); 2 Department of Surgery, School of Medicine, Nazarbayev University, Astana 010000, Kazakhstan; 3 Clinical Academic Department of Women’s Health, CF “University Medical Center”, Astana 010000, Kazakhstan; talshynu@yandex.ru; 4 Department of Obstetrics and Gynecology #1, NJSC “Astana Medical University”, Astana 010000, Kazakhstan; marat.a@amu.kz (A.M.);; 5 Department of Medicine, School of Medicine, Nazarbayev University, Astana 010000, Kazakhstan; 6 Department of Obstetrics and Gynecology #2, West-Kazakhstan Marat Ospanov Medical University, Aktobe 030000, Kazakhstansveta-r.mail.ru@mail.ru (S.R.); 7 Department of Medicine #3, NJSC “Astana Medical University”, Astana 010000, Kazakhstan; 8 Faculte’ des Sciences de Tunis, Universite’ de Tunis El Manar, Tunis 5000, Tunisia; 9 Clinical Academic Department of Internal Medicine, CF “University Medical Center”, Astana 010000, Kazakhstan

**Keywords:** miscarriage, pregnancy loss, recurrent pregnancy loss, recurrent miscarriage, RPL, RPL management

## Abstract

Recurrent pregnancy loss is a complex health challenge with no universally accepted definition. Inconsistency in definitions involves not only the number of spontaneous abortions (two or three) that are accepted for recurrent pregnancy loss but the types of pregnancy and gestational age at miscarriage. Due to the heterogeneity of definitions and criteria applied by international guidelines for recurrent pregnancy loss, the true incidence of recurrent miscarriage, which is reported to range from 1% to 5%, is difficult to estimate. Moreover, the exact etiology of recurrent pregnancy loss remains questionable; thus, it is considered a polyetiological and multifactorial condition with many modifiable and non-modifiable factors involved. Even after thoroughly evaluating recurrent pregnancy loss etiology and risk factors, up to 75% of cases remain unexplained. This review aimed to summarize and critically analyze accumulated knowledge on the etiology, risk factors, relevant diagnostic options, and management approach to recurrent pregnancy loss. The relevance of various factors and their proposed roles in recurrent pregnancy loss pathogenesis remains a matter of discussion. The diagnostic approach and the management largely depend on the etiology and risk factors taken into consideration by a healthcare professional as a cause of recurrent miscarriage for a particular woman or couple. Underestimation of social and health consequences of recurrent pregnancy loss leads to compromised reproductive health and psychological well-being of women after miscarriage. Studies on etiology and risk factors for recurrent pregnancy loss, especially idiopathic, should be continued. The existing international guidelines require updates to assist clinical practice.

## 1. Introduction

Spontaneous pregnancy loss is a common medical condition in reproductive-age women [1]. According to a worldwide estimation, 23 million cases occur annually [1].

Cases of early pregnancy loss are accepted by healthcare providers as an inevitable and unavoidable health issue, and the importance of the condition is misjudged [1,2]. Thus, international guidelines focus on recommendations for the diagnosis and management of repeated consecutive miscarriages only [3,4,5,6,7,8]. Moreover, investigations and treatment options vary internationally due to guidelines differences [9].

The situation is even more dramatic for women/couples with recurrent pregnancy loss (RPL) [10]. Even if the prevalence of RPL is not very high in the general population, for a single woman who suffers from pregnancy loss, it matters a lot [1]. Thus, each pregnancy loss case merits careful investigation to identify specific causative agents and risk factors [8].

In many cases, women do not clearly understand the cause of miscarriage and its recurrence [10,11]. The problem is underestimated as a simple physical health issue, which in most cases does not lead to serious health consequences. However, the psychological impact of the event is far more serious than the clinical presentation and subsequent physical harm [1,11]. Moreover, many societies, due to their cultural and traditional beliefs, determine women’s status based on their ability to conceive and give birth [12,13,14]. Thus, even in the 21st century, women who are incapable of childbearing could be treated with contempt and negligence, resulting in loneliness and stigmatization by family and society [1,12].

Considering the versatility of the health problem in RPL, an appropriate diagnostic approach to RPL and careful and optimal management that could prevent a recurrence is required [2,10,11,15]. This review aimed to summarize and critically analyze assembled knowledge on the etiology, risk factors, relevant diagnostic options, and management approach to recurrent pregnancy loss. A better understanding of RPL causes might result in revealing new insights for the prevention of repeated miscarriages.

## 2. Materials and Methods

A non-systematic literature review was conducted by the authors via searching exciting sources on RPL etiology, risk factors, diagnosis, and management. The literature search was performed in PubMed, Google Scholar, Scopus, Web of Science, and EBSCO databases up to 2023. The keywords, combinations of keywords, and MeSH IDs used for the literature search are reported in Supplementary Table S1. Based on the authors’ evaluation, the most pertinent to the subject of the investigation sources published in English have been read and used for the review. The results of the literature search have been presented logically to illustrate what has been reported on the topic of the discussion. Due to the nature of the findings, a narrative synthesis of the results from selected articles has been opted for. This work has some limitations: (1) only English language papers were included; (2) due to the heterogeneous nature of the studies included in this review (a diverse quality, study design, and outcomes assessed), only a narrative synthesis was possible.

## 3. Results and Discussion

### 3.1. Study Selection

**Figure 1:**
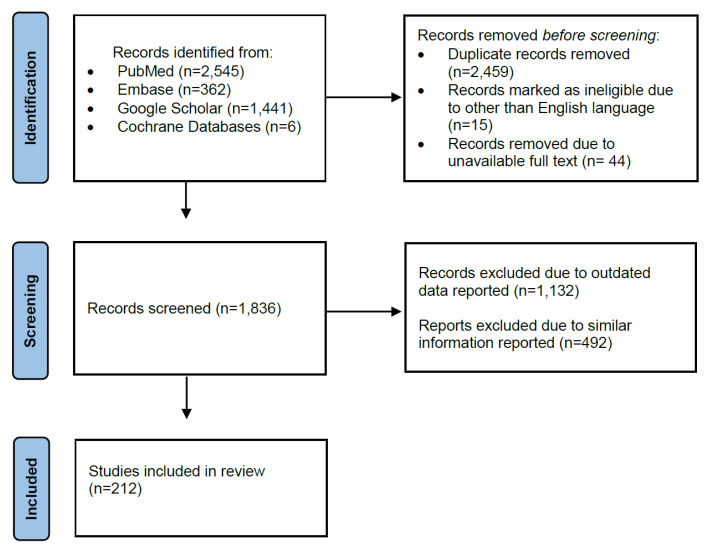
Studies selection flow-chart.

The initial screening using search strategy and keywords (Supplementary Table S1) on PubMed, Medline, Cochrane database, and Google Scholar identified 4354 articles. After the screening, 4142 articles were removed due to repeated records, the different language used (other than English), unavailable full texts, etc. (Figure 1). Finally, 212 most recent and reporting up-to-date data articles were included in this review.

### 3.2. Recurrent Pregnancy Loss Definitions and Terminology

Several national and international guidelines are available on RPL management. However, there is no consensus among them related to the number of pregnancies lost, the type of pregnancies reported (biochemical, clinical), the gestational age at pregnancy loss, and the sequence of previous pregnancy losses (Table 1) [3,4,5,6,9,15,16,17].

Back in 1976, the World Health Organization (WHO) defined the RPL as three and more consecutive miscarriages before the 22nd week of gestation or the loss of a fetus weighing <500 g [16]. Later in 2011, in line with the WHO definition, the Royal College of Obstetricians and Gynecologists (RCOG) guideline defined recurrent miscarriage as the loss of three or more consecutive pregnancies before 24 weeks of gestation [3]. However, without specification of the fetal weight (Table 1).

The definition of the European Society of Human Reproduction and Embryology (ESHRE) differs from the WHO and RCOG. The original ESHRE guideline defines RPL as the spontaneous loss of two or more pregnancies from the time of conception until 24 weeks of gestation [4,5]. This definition includes miscarriages both after spontaneous conception and assisted reproductive technology (ART). However, excludes cases of implantation failure, and ectopic and molar pregnancies [4], thus covering only clinically recognized pregnancies [9,18]. In the most recently released updated ESHRE guideline, no changes were implemented to the definition of RPL [ESHRE 2023].

Similar to the ESHRE definition, the American Society of Reproductive Medicine (ASRM) defines RPL as a disorder featured by the spontaneous loss of two or more clinical pregnancies [6,7]. In comparison with the ESHRE and ASRM definitions, the latest available guideline on RPL from the German Society of Gynecology and Obstetrics (DGGG), the Austrian Society of Gynecology and Obstetrics (ÖGGG), and the Swiss Society of Gynecology and Obstetrics (SGGG) follow the WHO definition, three and more consecutive recurrent miscarriages, for the purposes of recommendations on diagnosis and management [19].

From a clinical practice point of view, the ESHRE and ASRM guidelines are more beneficial for patients with RPL as they offer a special diagnostic approach to women after two consecutive pregnancy losses but not after three as recommended in the RCOG and DGGG/ÖGGG/SGGG guidelines (Table 1). Supporting this, in the ESHRE guideline, the importance of the RPL as a health issue and the necessity of further epidemiological investigations on the effect of different RPL definitions on diagnosis, management, and prognosis is recommended [4].

The terminology used to depict the type and pattern of spontaneous pregnancy loss is also inconsistent between international guidelines and researchers [18]. The following terms are used in the special literature: “recurrent pregnancy loss”, “recurrent miscarriage”, and “habitual abortion” [3,4,15,20]. The term “miscarriage” relates to an intrauterine embryo/fetal death confirmed by ultrasound or histology [21]. Thus, the ESHRE guideline recommends the term ‘recurrent pregnancy loss’ to describe repeated spontaneous pregnancy loss. In contrast, the term ‘recurrent miscarriage’ is suggested to be reserved for the recurrent loss of confirmed intrauterine pregnancies [4]. Therefore, non-visualized biochemical pregnancy losses and failed pregnancies of unknown localization should be differentiated from miscarriages [18,21].

Gestational age at pregnancy loss leads to even more serious debates as various national and international guidelines while defining RPL refer to 20, 22, or 24 weeks of gestation or fetal weight <350 g or <500 g if the gestational week is unknown [18,19].

A very limited number of studies investigated whether the consecutive or non-consecutive nature of recurrent miscarriage plays a role in the prognosis of the following pregnancies and for live birth [17,22,23]. In these studies, the sequence of pregnancy losses is proposed to be a predictor for future conception outcomes. Results of two studies [17,22] revealed the absence of significant difference in outcomes for consecutive or non-consecutive RPL and whether the patient had a live birth in the past. The study by Egerup et al. [23] reported that delivery in women with secondary RPL “eradicates the negative prognostic impact” of previous miscarriages. The authors concluded that only consecutive pregnancy losses should be counted for the definition of RPL [23]. This is an important finding; however, it should be supported by more studies with a larger sample size.

As the guidelines on RPL and their stated definitions indicate when certain diagnostic work-up should be considered for patients with recurrent miscarriage to improve clinical care and management of women with RPL, a consensus on the definition should be reached by international societies for a more consistent risk assessment of an individual patient [17].

### 3.3. Epidemiology of Recurrent Pregnancy Loss

Nearly 10–15% of clinical pregnancies and 30% of all pregnancies terminate with spontaneous abortion, making it the most frequent pregnancy complication [6,15,24,25,26]. Most of the sporadic pregnancy losses before 10 weeks of gestation result from chromosome aberrations (monosomy, trisomy, and polyploidy) [1,6,24,27,28,29,30,31,32].

The variance and discrepancy in definitions of RPL lead to difficulty in the real prevalence estimation [4,18]. Moreover, cultural and traditional relationships may prevent women from having open discussions about their miscarriages due to the possible blame from the society she lives in [12,13,14,18]. Furthermore, RPL incidence may be underreported since not many countries must document pregnancy losses as a separate indicator in national healthcare databases [18]. All these factors contribute to the underestimation of RPL prevalence in some world regions.

Based on the available sources, it is estimated that around 5% of females could experience two or more consecutive miscarriages, and only 0.4–1% have three or more [3,4,5,6,18,33,34,35,36,37,38]. The risk for females to have a spontaneous abortion after a prior single miscarriage is 12–20%. After suffering two miscarriages, the risk rises to 29%, and after three—36% [3,39,40]. As stated in the RCOG guideline on RPL, previous successful delivery does not preclude a woman from developing recurrent miscarriages [3]. However, one of the recent studies reported that live birth in women with secondary RPL could alleviate the negative prognostic impact of previous miscarriages [23]. Thus, further studies are required to prove these contradicting findings.

### 3.4. Recurrent Pregnancy Loss Etiology and Risk Factors

**Figure 2:**
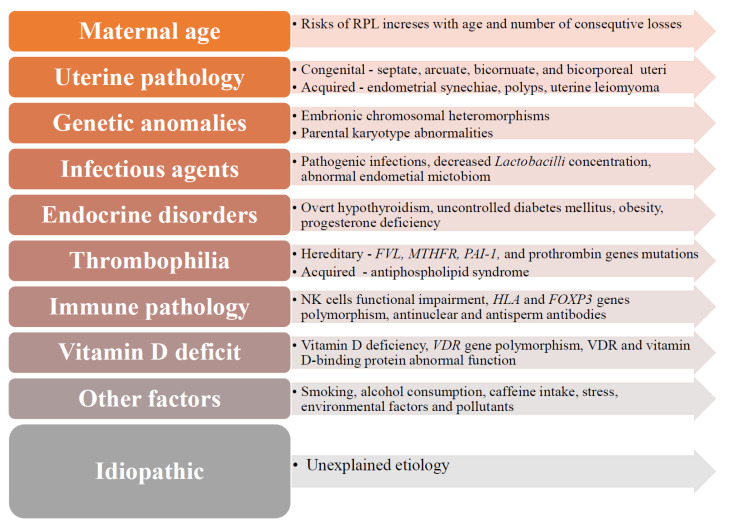
Etiology and risk factors of recurrent pregnancy loss. Figure legend: FOXP3—forkhead 3 box protein; FVL—factor V Leiden; HLA—human leukocyte antigen; MTHFR—methylenetetrahydrofolate reductase; NK—natural killer cells; PAI-1—plasminogen activator inhibitor-1; VDR—vitamin D receptors.

RPL is a polyetiological condition, and the reason is often unknown (Figure 2). Several factors have been suggested to contribute to RPL pathogenesis, including maternal age (9–75%), endocrine diseases (17–20%), uterine morphological pathologies (10–15%), chromosomal abnormalities (2–8%), thrombophilia, infectious agents (0.5–5%), and autoimmune disorders (20%) [1,24,28,29,30,31,41]. Nevertheless, in approximately 50–75% of RPL cases, the exact cause is not clearly identified and, therefore, remains unexplained (idiopathic) [15,42,43,44,45].

#### 3.4.1. Maternal Age

Women’s age at conception is reported to serve as an independent risk factor for miscarriage [18,46,47,48,49]. The risk of miscarriage is slightly elevated among young mothers and then increases abruptly in advanced-age mothers [46,49]. According to the RCOG guideline data, the age-related risk of pregnancy loss is 13% in ≤19 years; 11–12% in 20–29 years; 15% in 30–34 years; 25% in 35–39 years; 51% in 40–44 years; and 93% in ≥45 years age groups [3,15]. According to the DGGG/ÖGGG/SGGG guideline, the age-related risk of recurrence is: (1) after two miscarriages increase from 24% at 25–29 years up to 44% at 40–44 years; (2) after three and more miscarriages increase from 42% at 25–29 years up to 65% at 40–44 years [19]. The increased risk of miscarriage for women >35 years old appears even more dramatic, considering that the chances to conceive in this age group decline with years [46,47,48,49].

#### 3.4.2. Uterine Factors

The contribution of uterine structural anomalies to the etiology of RPL was reported in several studies and found to be present in about 7–28% of women with RPL compared with 4–7% of women in the general population [11,18,50].

The most common congenital uterine anomalies include septate uteri, arcuate, and bicorporal uteri (Figure 2) [50]. Among patients with congenital uterine defects, women with a septate uteri have the highest incidence of recurrent miscarriage—44.3%, patients with bicornuate uteri—36%, and arcuate uteri—25.7% [2]. Congenital genital tract anomalies are associated with late 1st-trimester and 2nd-trimester pregnancy losses, rarely with early pregnancy losses [18].

Acquired uterine structural defects such as submucosal uterine leiomyomas, endometrial synechiae, and polyps interfere with the process of implantation and embryo development, thus, may result in recurrent miscarriage [15,50]. These conditions are associated with 6–15% of RPL [15,50,51].

#### 3.4.3. Genetic Factors

A lot has been reported on the role of genetic factors in RPL, as chromosomal abnormalities are one of the significant causes of miscarriage in the first trimester of pregnancy [45]. The contribution of genetic predisposition to the altered risk of RPL is based on three lines of evidence. First, family studies confirmed that siblings of women with RPL are at a higher risk of RPL compared to ethnically-matched control women [52]. Second, the risk of RPL is highest in subjects carrying specific at-risk genetic variants [53]. Third, RPL is likely to develop in the first trimester of gestation.

Several genetic factors linked with RPL were identified. These include DNA methylation, sperm DNA fragmentation, chromosome heteromorphisms, and single nucleotide genetic variation [53,54,55,56]. However, none was proven to be a stand-alone risk factor for RPL.

Well-known genetic causes of RPL are gross chromosomal defects and variations of allelic expression [57]. At least 50–60% of all sporadic miscarriages are associated with cytogenetic abnormalities [3,18,54,58]. Significant overlaps are present between the genetic causes of sporadic and RPL. However, RPL could occur even in cases of normal embryonic genetic profile [2,59]. The frequency of karyotype abnormality affects approximately 2–8% of couples with RPL [2]. Balanced reciprocal translocations and Robertsonian translocations are reported for 2–5% of couples with RPL [6].

The inactivation of X-chromosomes that could occur during early embryogenesis has been proposed as a possible cause of recurrent miscarriage [60]. A case-control study, which compared skewed X-chromosome inactivation (XCI) status between patients with RPL and healthy women, found an extremely skewed XCI (>90%) in 17.7% of women with recurrent miscarriage. In comparison, this indicator was as low as 1.6% in controls [60,61].

Genetic risk factors, including abnormal embryonic genotypes and parental chromosomal rearrangements, could be a background for more than 50% of RPL cases [49]. Apart from karyotype abnormalities, genetic variants can influence tissue development in pregnancy. For example, some studies reported polymorphisms in vascular endothelial growth factor (VEGF)-related genes that could be associated with RPL [62,63].

#### 3.4.4. Endocrine Disorders

Endocrine disorders play a significant role in approximately 12–20% of RPL [1,2,18,25]. Although systemic maternal endocrine diseases such as diabetes mellitus and thyroid pathologies have been associated with spontaneous abortions [2,3,11,64], the RCOG guideline suggests that “well-controlled diabetes is not a risk factor for recurrent miscarriage” [2,3], while poorly controlled diabetes with high levels of HbA1c is [15].

Even though subclinical hypothyroidism does not increase the risk of RPL [3], clinically recognizable hypothyroidism cases with moderate and significant elevated thyroid-stimulating hormone (TSH) is a well-known risk factor for miscarriage [11,18,64] and impaired fetal and newborn development [64,65].

Since progesterone plays a major physiologic role in the process of successful implantation and pregnancy development [3,10], insufficient progesterone levels (i.e., luteal phase deficiency) are assumed to be associated with spontaneous pregnancy loss [2,18,66]. As a part of pregnancy follow-up, patients with a history of recurrent miscarriage are tested for luteal phase defect via serial investigations of serum progesterone concentration [2]. However, attempts to identify the specific pathologic patterns in short luteal in women with RPL did not result in any evidence [18,66,67].

Polycystic ovarian syndrome (PCOS) is not considered a predictive factor for RPL [19]. However, obesity itself or related to PCOS increases the risk of recurrent miscarriage. Recent studies have reported that obesity in women with a previous history of RPL raises the risk of recurrent miscarriage [3,18,68,69].

#### 3.4.5. Infections

Severe infection of any site could potentially cause spontaneous abortion and late pregnancy complications [3,70]. Bacterial vaginosis in the 1st trimester of pregnancy has been shown as a risk factor for late miscarriages (after 14th weeks of gestation) and preterm delivery [3]. However, the role of infection in first-trimester RPL remains unclear [3]. Theoretically, a potentially harmful infection should persist in a woman’s body for the period of repeated consecutive pregnancy loss cases [3]. Studies investigating a direct association between Ureaplasma, Chlamydia, Mycoplasma, and Toxoplasma with RPL do not show strong evidence [3,15]. However, anti-inflammatory cytokines released by placental and decidual tissues in response to infections may lead to pregnancy loss. A recent study by Baqer et al. (2022) reported that certain interleukins (IL), such as IL-3, IL-17A, and IL-27, as the maternal immune response to infections could lead to abortion [71].

Many studies suggested chronic endometritis as a potential cause of RPL [72,73,74,75]. In the study by McQueen et al. (2021), a significantly higher rate of chronic endometritis in women with RPL was found [72], thus, supporting a link between chronic endometritis and recurrent miscarriage.

Apart from the chronic endometritis caused by pathogenic flora, currently, the endometrial cavity microbiome is considered an important predictor of success in pregnancy, no matter if it is induced or spontaneous [76,77,78,79,80]. The diverse bacterial populations in the endometrial lining in women with idiopathic RPL, particularly in the Lactobacillacae species, were reported by Masucci et al. (2023) [79]. Dysbacteriosis in the female reproductive tract is associated with recurrent miscarriage and should be considered a novel risk factor for RPL [78]. A recent study by Shi et al. (2022) identified that uterine endometrial microbiome analysis for women with a history of pregnancy loss before pregnancy may identify variants of microbiota associated with RPL [76]. This study demonstrated increased Ureaplasma species in the uterine endometrial microbiome of women with RPL, which is found to be a risk factor for miscarriage. Thus, researchers suggest that different compositions of vaginal-endometrial microbiota could be classified based on bacterial patterns and association with RPL, which would allow a personalized diagnosis and approach based on the microbiota composition [79].

#### 3.4.6. Thrombophilia

Thrombophilia and the predisposition to improper coagulation can affect chorionic blood flow and cause vasculopathy leading to pregnancy loss [81,82,83]. This assumption is confirmed by the recent meta-analysis of 89 studies with 30,254 participants involved, which suggested that hereditary thrombophilia is associated with RPL [84]. The most prevalent types of thrombophilia associated with RPL are hereditary (factor V Leiden, genetic polymorphism of methylenetetrahydrofolate reductase (MTHFR) enzyme, prothrombin gene mutation, protein C deficiency, etc.) or acquired (antiphospholipid syndrome (APS)) [3,83].

Mutations in the factor V Leiden gene (FVL, G1691A) and prothrombin gene (PG, FII, G20210A) are considered risk factors for RPL [85]. FVL is the most common genetic thrombophilia, with an estimated prevalence of 1–10% [86], which may vary in different populations. A prevalence of 5–9% was reported among the European population, while the mutation is almost absent in African and Asian populations [83,87,88]. The variations in FVL mutation prevalence among distinct ethnic groups dictate the difference in risk of RPL associated with FVL mutation [84]. Acknowledging the stratification by geographical location, positive associations between FVL mutation and RPL were found in studies conducted in Africa, the Middle East, Europe, and Asia [84]. Heterozygous FVL mutation carrier women are not at increased risk for early fetal loss [83]. However, according to reports, the FVL mutation carrier state may increase the susceptibility to recurrent miscarriage [86,89].

The prothrombin gene (PG) mutations contributing to RPL were found among 2–4% of European Caucasians, less frequently among women of African and Asian inheritance [90]. Similar to the FVL mutation, women who are heterozygous for prothrombin mutation G20210A are not at increased risk for early pregnancy loss [83]. Overall, FVL and PG mutations may increase the risk of RPL by 2.44-fold and 2.08-fold, respectively [84].

Researchers also reported a role plasminogen activator inhibitor-1 gene (*PAI-1*, also known as *SERPINE1*) and found a significant difference between RPL and the control group for *PAI-1* 4G/5G mutation and *PAI-1* 4G/4G mutation variants [91,92]. The authors concluded that patients with three or more abortions had a higher ratio than those with two abortions (*p* < 0.05).

Studies also report that factor XIII (FXIII) mutations could affect the physiology of fibrinolysis and increase the risk of RPL in women homozygous for the FXIII Val34Leu [93,94]. It is supported by a meta-analysis of Jung et al., (2017), which reported a link between *F13A1* Val34Leu polymorphism and recurrent miscarriage [93]. The researchers found significant associations between *F13A1* Val34Leu mutations and the risk of RPL in Asian populations; however, the association between Europeans and South Americans was insignificant [93].

Vascular endothelial growth factor (VEGF) is an important angiogenic factor that plays a crucial role in the process of embryo implantation [95]. Researchers analyzed the *VEGF*, *VEGFR-1*, and *VEGFR-2* genes expression and found that women with RPL had a lower level of VEGF and higher levels of VEGFR-1 and VEGFR-2 levels in the endometrium if compared to healthy controls [95,96]. A meta-analysis by Xu et al. (2015) identified *VEGF* polymorphisms (rs1570360, rs3025039, rs2010963, and rs3025020), which were associated with an increased risk of RPL [95,97].

The prevalence of the MTHFR 677C > T differs depending on location and ethnic background [98,99,100]. The MTHFR 677C > T mutation has been found to be higher among Italian and Spanish populations in Europe and lower in Germans and African Americans [98,100]. Among Caucasians living in Australia, Brazil, Canada, and the USA, the homozygous mutation is reported in up to 15% of the population [98,100,101]. Little data are available about the Asian population. Polymorphism of the MTHFR gene at position 677C > T has an impact on the function of the MTHRF enzyme in the homocysteine metabolism [101,102,103]. In turn, elevated plasma homocysteine levels have been proven to serve as a risk factor for infertility and pregnancy complications such as miscarriage and preeclampsia [101,104,105,106]. An association of MTHFR 677C > T and RPL has been reported by researchers [107,108].

APS is an autoimmune condition featured by antiphospholipid antibody formation and associated with thrombotic events and pregnancy complications, including RPL [2,3,18,109,110]. The prevalence of antiphospholipid antibodies is estimated at 15–20% among women with RPL [2,3,111,112], while in low-risk women, this indicator is less than 2% [3]. Moreover, in women with RPL associated with APS, the live birth rate was reported to be low (10%) if no pharmacological management was applied [3]. The APS causes an inflammatory response to antiphospholipid antibodies on vascular endothelium and chorionic/placental cells, which promotes thrombosis [18].

Many studies demonstrated a strong association between APS and adverse pregnancy outcomes (RPL, stillbirth, preeclampsia) [2,3,83,109,113,114]. According to the RCOG guideline as an adverse pregnancy outcome associated with APS, the following conditions have been highlighted: (1) history of three or more consecutive spontaneous abortions before 10 weeks of gestation; (2) history of one or more pregnancy losses after the 10th week of gestation with morphologically normal fetus; (3) history of one or more preterm delivery before the 34th week of gestation due to placental disease [3]. Thus, specific attention to women with the listed conditions must be drawn to prevent further pregnancy losses and other associated complications.

#### 3.4.7. Immune Factors

The immune response control is important for a successful pregnancy and related to the link between genetic variants and increased risk of RPL [83,112,115]. Genetic variants of the human leukocyte antigen (HLA) system and difference in immune tolerance has been proposed to contribute to RPL as they lead to the suppression of immune regulators, invigoration of inflammatory processes, and immune rejection [27,34,37,112,116,117,118].

During physiologic pregnancy, the female systemic immune response is modulated in such a way that a decreased cell-mediated immunity occurs to let the semi-allogenic fetus develop in the uterine cavity [83,119,120]. Dysfunction of these elements could lead to pregnancy loss [121,122].

Immune mechanisms play a significant role in the pathogenesis of recurrent miscarriage. There is a strong association between HLA alleles and autoimmune diseases [79,80]. It has been stated by many authors that the majority of idiopathic RPL is to be due to immunogenetic etiology such as HLA gene variations [42,58,115,123,124]. However, the exact connection between HLA and specific diseases is not fully understood yet, as many complementary genetic factors and environmental influences may significantly contribute to the pathologic process [125].

In this view, the HLA system is the most important immune factor in pregnancy maintenance and might play a crucial role in the incidence of RPL. There are several well-known autoimmune conditions that could contribute as immunologic causes of RPL: systemic lupus erythematous, APS, rheumatoid arthritis, inflammatory bowel and celiac diseases, anti-thyroid, anti-nuclear and anti-sperm antibodies [2,79,80,126]. Moreover, there are also alloimmune causes that have been proposed [71,112].

It has been reported that unexplained/idiopathic recurrent miscarriage is associated with the presence of specific maternal HLA alleles and with the degree of HLA mismatching between mother and child [42,112]. Through the years, several researchers investigated the association of specific maternal HLA class II alleles (DR, DQ, and DP) with RPL incidence [34,52,127,128,129,130]. Results of the Japanese researchers’ investigation revealed that HLA-DPB1*0402 and DPB1*04 alleles were found to be significantly increased in the study group compared with the healthy fertile women [127]. The results of the other researchers propose an association of the DQB1*03 and DRB1*03 alleles with RPL [129,130].

The effect of HLA genes on the intestinal microbiome has already been defined [79]. The prevalence of HLA-DQ2/DQ8 positivity in women suffering from RPL is around 53% and occurs twice more often than in the general population [79,80]. According to the most recent study, the HLA DQ2/DQ8 positive-RPL and HLA DQ2/DQ8 negative-RPL women revealed different endometrial and vaginal microbiota compared to healthy women [79]. Thus, HLA class II allele polymorphisms could be a risk factor for RPL via different paths.

Recently the role of forkhead 3 box protein (FOXP3) in RPL was proposed [33,131,132]. FOXP3 is a nuclear transcription factor required to induce immunosuppressive activity [131,133]. Having strong immunosuppressive properties in regulatory T cells (Treg), FOXP3 may have an immunosuppressive impact in trophoblastic cells [131,133]. Thus, this feature of FOXP3 may serve as a mechanism of maternal tolerance to semi-allograft embryos. This hypothesis proposing the role of Treg in RPL pathogenesis is supported by recent research findings, which suggest that FOXP3 gene variants and haplotypes could be associated with RPL [33,132].

#### 3.4.8. Vitamin D Deficiency

Vitamin D deficiency is a growing global health concern. Owing to its pleiotropic biological effects, vitamin D insufficiency contributes to the pathogenesis of vascular diseases, neoplastic processes, and degenerative diseases of the nervous system [134,135]. An association between low vitamin D levels and adverse maternal and neonatal pregnancy outcomes was demonstrated by studies on many ethnic groups [136,137,138,139,140]. Many lines of evidence implicate defective vitamin D activity with a heightened risk of RPL [138,141].

Vitamin D maintains its function through vitamin D receptors (VDR), which polymorphism was associated with spontaneous preterm birth in Northeastern Brazilians [142,143,144]. Furthermore, a role for VDR and signaling pathways in the placenta was proposed [142], and reduced VDR expression was seen in the chorionic villi and decidua in women with RPL compared with control women [142,145]. The presence of VDR polymorphisms might lead to abnormal function of VDR and subsequent problems in the vitamin D-mediated metabolic processes [81]. VDR expression by epithelial and stromal cells in the endometrium and its increased levels in pregnancy confirm the central role of Vitamin D in the maintenance of normal pregnancy [138,146,147,148,149].

However, the exact role of vitamin D in pregnancy failure remains controversial. While some studies demonstrated an association of RPL with decreased vitamin D levels, lower expression of VDR, or lower levels of 1α-hydroxylase [135,138,141,145,146], others reported no association between vitamin D deficiency and pregnancy failure [136,150,151,152].

#### 3.4.9. Other Risk Factors

Other risk factors for RPL include stress, alcohol, smoking, ethnicity, a history of previous miscarriage and preterm birth, and environmental factors [11,24,153,154,155,156].

The strong association between cigarette smoking and poor pregnancy outcomes (stillbirth, intrauterine growth restriction, placenta previa, preterm labor, and congenital anomalies) was reported by Toth et al. (2018) [19]. Other studies found that nicotine consumption considerably increased the risk of RPL within the general population [157,158].

There were limited studies investigating the link between coffee consumption and the risk of RPL [159,160]. Some studies reported a dose-dependent association between coffee intake and pregnancy loss, as caffeine intake might increase the risk of RPL [19,159]. However, a recent meta-analysis on female caffeine intake and its relation to the risk of RPL did not find a significantly increased risk of RPL in the general population [156].

Researchers also suggest that psychological stress and stressful events during pregnancy could be associated with an increased risk of spontaneous abortions [11,19,161]. Although a lot is known about the causes of RPL, many important questions regarding the etiology and risk factors of RPL remain unanswered, and the origin of RPL is complex and poorly understood.

### 3.5. Diagnostic Approach to Recurrent Pregnancy Loss

**Figure 3:**
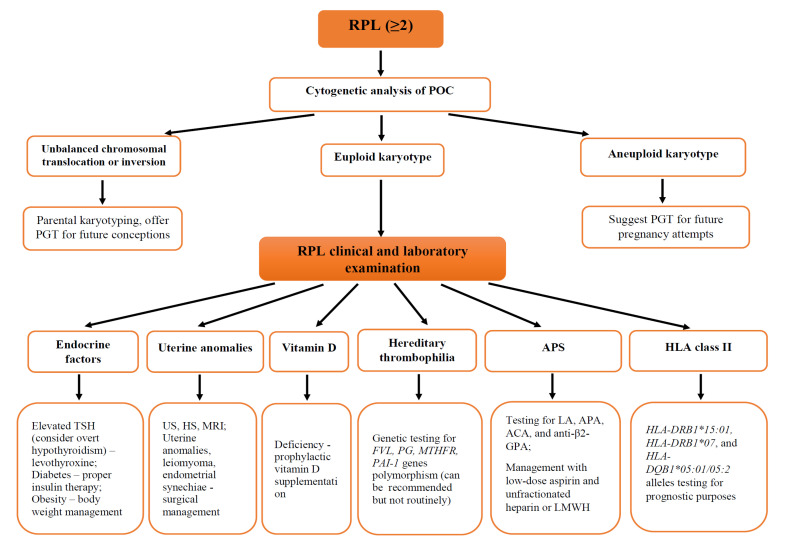
Recurrent pregnancy loss diagnostic and management algorithm. Figure legend: LA—lupus anticoagulant; ACA—anticardiolipin antibodies; anti-β2-GPA—anti-β2 glycoprotein antibodies; APA—antiphospholipid antibodies; APS—antiphospholipid syndrome; FVL—factor V Leiden; HLA—human leukocyte antigens; HS—hysteroscopy; MI—minimally invasive; MTHFR—methylenetetrahydrofolate reductase; LMWH—low-molecular-weight heparin; POC—products of conception; PAI-1—plasminogen activator inhibitor-1; PG—prothrombin gene; PGT—preimplantation genetic testing; RPL—recurrent pregnancy loss; TSH—thyroid-stimulating hormone; US—ultrasound.

For a proper diagnostic approach, a careful past medical history of patients with RPL and identification of etiological and risk factors should be performed (Figure 3). The evaluation strategy for women with a recurrent miscarriage should be focused on those etiological and risk factors that could be modified and, thus, the patient could be effectively treated [10,18]. Due to the multiple potential etiological and risk factors (Figure 1) that might be associated with RPL, women/couples suffering from RPL should be evaluated by a multidisciplinary team. Genetic factors, maternal age, immune disorders, and hereditary thrombophilia comprise a group of non-modifiable factors. However, available contemporary management options could be applied as necessary.

#### 3.5.1. Prognostic Tools for Recurrent Pregnancy Loss Prediction

The development of prognostic tools for the prediction of pregnancy loss recurrence and the live birth rate in cases with repeated pregnancy loss could improve the management of women with RPL [162]. In addition, prediction tools might assist in providing a prognosis for couples with RPL [162].

In a recent study by Bashiri et al. (2023), researchers found that the live birth rate in women with RPL was significantly associated with age, number of previous miscarriages, primary and secondary RPL, and positive RPL-related workup [162]. Based on these factors taken into consideration, two scoring prediction models were created by the researchers, which showed an increase in the live birth rate with rising scores [162].

In another study, Chinese researchers have made efforts to develop a predictive scoring system for RPL [163]. Through the multivariate analysis of risk factors for spontaneous pregnancy loss, the researchers identified and included in the RPL scoring system model the following adverse risk factors: antiphospholipid antibodies, antinuclear antibody spectrum, and protein S deficiency [163]. Each of these factors contributed 1 point to the risk probability. This scoring system is proposed by the authors for accurate prediction of the recurrent miscarriage risks and could be useful in the identification of appropriate risk-related interventions to decrease RPL incidence [163]. However, the efficacy of the scoring system should be tested in clinical practice by applying it to a large cohort of patients as was previously performed for such algorithms developed for other gynecological conditions [164].

According to the updated 2022 ESHRE guideline, women’s age, together with precise and complete pregnancy history, are important in predicting the live birth chances in the next pregnancy [5]. Therefore, the recent ESHRE recommendations suggest setting up a prognosis based on the woman’s age, “complete pregnancy history, including a number of previous pregnancy losses, live births, and their sequence” [5].

#### 3.5.2. Genetic Factors Identification

Couples experiencing RPL should have karyotyping performed to detect structural chromosomal anomalies that could be responsible for recurrent miscarriages [3,6]. In addition, RCOG and ASRM guidelines recommend cytogenetic analysis of the products of conception (POC) (Figure 3) [3]. However, the original ESHRE guideline is more skeptical about the value of routine karyotyping of parents and POC [4,15], as karyotyping procedures in the current pregnancy might lead to complications. Another novel available option is preimplantation genetic testing (PGT), usually used for women seeking ART [45]. PGT allows the testing of a few embryo cells and the selection of an embryo without genetic abnormalities [9]. Since aneuploidy is the most common embryonic chromosomal abnormality causing pregnancy loss, patients with RPL with reported previous embryonic chromosomal abnormalities could be offered [45]. However, the risk of PGT should not outweigh the potential benefit from the procedure. Moreover, even if this diagnostic option is available, PGT is not recommended for patients with RPL [4].

Moreover, according to the most recent ESHRE recommendations, the evaluation of sperm DNA fragmentation in couples with recurrent miscarriages should be considered [5].

#### 3.5.3. Uterine Anomalies Diagnosis

In cases of RPL caused by congenital structural pathology of the uterus, ultrasound (US) evaluation with two-dimensional and three-dimensional modalities applied is recommended [3,15]. Moreover, acquired genital pathologies such as uterine leiomyomas and adenomyosis should also be considered. Thus, the recent ESHRE guideline highlights the association of adenomyosis with higher rates of pregnancy loss and recommended to perform two-dimensional US to exclude adenomyosis [5].

To confirm the diagnosis of uterine anatomic pathologies suspected on US examination, further investigations could require further assessment using hysteroscopic or laparoscopic equipment [3]. Pelvic magnetic resonance imaging (MRI) could be helpful in complex cases, however, is not routinely necessary [50].

#### 3.5.4. Chronic Endometritis Assessment

Although there is evidence of the role of chronic endometritis in the pathogenesis of RPL, the ESHRE, ASRM, and RCOG guidelines do not yet recommend endometrial biopsy in the workup for RPL [3,4,5,6,72]. Only the DGGG/ÖGGG/SGGG guideline considers endometrial biopsy for women with RPL to exclude chronic endometritis [19].

In the recent study by McQueen et al. (2022), the authors suggest a value of a pathologic evaluation for chronic endometritis, which should be performed for all patients who undergo hysteroscopic resection of the retained chorionic tissue RPT following miscarriage [73]. Office hysteroscopy is suggested as a useful diagnostic tool in such cases [73,75]. However, less invasive biopsy methods are available for endometrial biopsy [165]. Additional research is needed to determine if endometrial biopsy in patients with RPL could contribute to the improvement of their management and prevention of future pregnancy losses [73].

#### 3.5.5. Endocrine Factors Evaluation

For women with two or more spontaneous abortions associated with endocrine pathologies, TSH, thyroid hormone levels, and thyroid antibodies should be tested [6,15]. However, since the role of luteal phase deficiency in RPL is uncertain, routine testing for progesterone levels is not recommended.

#### 3.5.6. Thrombophilia Assessment

Although case-control studies found an association between hereditary thrombophilia and pregnancy loss, ESHRE and ASRM do not recommend routine screening for MTHFR, FVL, PG, and other mutations associated with thrombophilia and RPL, and only women with venous thromboembolism (VTE) and a history of recurrent miscarriage should be tested for inherited thrombophilia [2,3,4,5,6]. Some studies suggest considering the test for FVL mutation in patients with unexplained/idiopathic early RPL [89].

Monitoring plasma coagulation markers during pregnancy is not recommended for females with a history of RPL. Furthermore, as stated in the DGGG/ÖGGG/SGGG guideline, these markers “must not be used as an indication to initiate therapy to prevent miscarriage” [19].

For APS, acquired thrombophilia, the diagnostic criteria are well defined and require the investigation of lupus anticoagulant, antiphospholipid, anticardiolipin, and anti-β2 glycoprotein antibodies [2,3,4,5,6,166].

#### 3.5.7. Immune Factors Evaluation

Owing to the relative novelty of the theories on the role of immune factors in RPL and inconsistent research evidence in this field, there is no consensus on the necessity of laboratory workup for immune factors in women with RPL [5,19,112]. Out of all available international guidelines, only the DGGG/OEGGG/SGGG and updated in 2023 ESHRE guideline recommend examination of autoimmune factors. In case of a history of recurrent miscarriage, the DGGG/OEGGG/SGGG recommends assessing natural killer (NK) cells, Treg cells, and HLA genes [19,167]. In the updated ESHRE recommendations, a minor modification was made to the screening of the HLA system, namely only HLA class II (HLA-DRB1*15:01, HLA-DRB1*07, and HLA-DQB1*05:01/05:2 alleles) testing is suggested in “in very specific and defined circumstances” for prognostic purposes [5].

#### 3.5.8. Vitamin D Levels

According to the Clinical Practice guidelines of the Endocrine Society, vitamin D deficiency is depicted as 25-hydroxyvitamin D serum levels <20 ng/mL, while vitamin D insufficiency is defined as 25-hydroxyvitamin D serum levels between 21 and 29 ng/mL [134,147,168,169]. Although researchers report that Vitamin D deficiency and insufficiency are associated with spontaneous abortions [138], there are no clear recommendations in the guidelines about the necessity of Vitamin D measurement as a part of preconception counseling or in the work-up plan for women with RPL.

### 3.6. Management of Recurrent Pregnancy Loss

Management of the accomplished pregnancy loss depends on the symptoms and is either surgical (uterine curettage/vacuum aspiration), medical (mifepristone and misoprostol), or expectant [1,10,170,171]. However, the story does not end up with the evacuation of conception products from the uterine cavity. Contrary, the struggle with RPL begins as the risk of subsequent miscarriages increases.

Although many guidelines and articles are published on the topic, healthcare providers still have queries about the optimal management and care plan for patients with RPL (Figure 3) [1,10]. Management and treatment options should be defined based on the etiology and risk factors identified during the diagnostic process.

#### 3.6.1. Uterine Anomalies Management

A limited number of studies evaluated the efficacy of uterine anomalies surgical treatment for RPL management, aiming prevention of further recurrent miscarriages. Published research data on surgical indications in cases of congenital and acquired uterine structural defects remain controversial [18,50,172].

Only one small randomized controlled trial (RCT) evaluated the benefits of surgical management of congenital uterine abnormalities on pregnancy outcomes [172]. This RCT concluded that hysteroscopic uterine septum resection “does not improve reproductive outcomes in women with a septate uterus” [172]. Thus, since the data are limited and no benefit from this invasive procedure for the reduction of pregnancy loss rates, the most recent ESHRE guideline is neutral about hysteroscopic uterine septum resection [5].

There are some results of retrospective studies confirming the positive effects of surgical treatment (removal of acquired uterine anomalies—leiomyomas, adhesions, or polyps) that may result in the reduction of pregnancy loss risks [18,50]. Thus, some authors suggest that submucosal fibroids, uterine synechiae, and endometrial polyps could be managed by hysteroscopic resection [15,50]. Moreover, some studies report decreased RPL rates in patients after uterine septum resection compared with untreated ones [173].

However, the ESHRE guideline stated that there is no sufficient evidence to recommend the hysteroscopic removal of submucosal uterine leiomyomas in women with recurrent miscarriages [4,50]. Furthermore, the RCOG guideline also reports insufficient evidence to assess the effect of congenital uterine septum resection in women with RPL due to septate uterus [3].

Despite these inconclusive data, some researchers suggest resectioning uterine septa, endometrial synechiae, and submucosal leiomyomas for patients with RPL to prevent further pregnancy losses [50]. Some other currently available minimally invasive approaches to uterine leiomyoma treatment could be considered as a fertility-sparing approach to women with RPL due to uterine leiomyoma [174].

#### 3.6.2. Management of Recurrent Pregnancy Loss Associated with Genetic Factors

Genetic counseling should be offered to couples with an abnormal parental karyotype for their awareness of a prognosis for the risk of future pregnancy losses [3,4,5,6]. Reproductive options for couples with chromosomal rearrangements include a natural pregnancy with/without PGT, gamete donation, and adoption [3]. PGT can be considered a treatment option for couples with abnormal parental karyotypes to select embryos without genetic pathologies [9]. This is usually performed as a part of treatment with ART. However, while considering ART, future parents should be made aware of a probability of 50–70% of a healthy live birth in the future with natural conception. In the case of in vitro fertilization (IVF) with PGT, this chance is approximately 30% [3]. Thus, the RCOG guideline concluded that PGT with IVF as a management option for women with genetic causes of RPL does not improve live birth rates [3,173].

#### 3.6.3. Progesterone Therapy

Progesterone and the physiologic function of progesterone receptors play a considerable role in early pregnancy development and thus, progesterone deficiency is assumed to be responsible for a proportion of miscarriages [67,175]. The well-accepted RCOG guideline states, “there is insufficient evidence to evaluate the effect of progesterone supplementation in pregnancy to prevent a miscarriage in women with recurrent miscarriage” [3]. Some studies reported that micronized progesterone supplementation for women with RPL “makes little or no difference to the live birth rate when compared with placebo” [176], and progesterone therapy did not result in a significant improvement in the rates of live births among women with the risk of miscarriage [67,175]. Moreover, the latest Cochrane database meta-analysis based on the available evidence suggested that progestogens probably do not influence the live birth rate for women with RPL [176]. Thus, there is still uncertainty over the effectiveness and safety of alternative progestogen treatments for recurrent miscarriages [176].

However, other studies conducted in this field reported contrary data supporting progesterone administration for RPL management [67,177,178,179]. One of the recent reviews that analyzed available clinical trials on the effect of vaginally administered progesterone prescribed in early pregnancy for the prevention of RPL reported an improvement in live birth rates in a subgroup of women with a history of recurrent miscarriages and bleeding [18,67]. As concluded by the authors, patients with a history of pregnancy loss who are experiencing bleeding in early pregnancy may benefit from the administration “of vaginal micronized progesterone 400 mg twice daily” [67].

Other studies investigated the dydrogesterone effect on RPL management [177,178]. A study by Arab et al. (2019) reports evidence of the dydrogesterone therapy effect in the reduction of pregnancy loss rate and recommends oral dydrogesterone (10–20 mg daily until the 20th week of gestation) for patients with idiopathic RPL [177]. Moreover, the most recent study by Bashiri et al. (2023) concluded that dydrogesterone treatment is associated with an increased live birth rate in women with RPL [178].

Thus, based on the recent research results [67,177,178,179] and the updated ESHRE guideline [5], vaginal progesterone could be suggested for the management of women with a history of three and more recurrent miscarriages [5].

The discrepancy in the evidence of the progesterone supplementation benefits for women with recurrent miscarriages and existing controversies in recommendations require more investigations to be conducted in this field.

#### 3.6.4. Thyroid Hormone Replacement

Based on ESHRE recommendations, euthyroid women with thyroid antibodies and RPL do not require thyroid hormone replacement therapy [5]. However, women with recurrent miscarriages and apparent clinical hypothyroidism diagnosed before or during early pregnancy should be offered levothyroxine [18,19]. The levothyroxine treatment improves pregnancy outcomes in patients with a history of RPL due to overt and subclinical hypothyroidism [111,112].

#### 3.6.5. Management of Thrombophilia

Many studies investigating pregnancy outcomes in patients with thrombophilia and RPL reported a similar approach to the use of anticoagulation therapy for the majority of thrombophilia types: FVL mutation, PG mutation, and APS [3,86,180]. For patients with RPL associated with FVL mutation, low-dose aspirin, low-molecular-weight heparin (LMWH), and low-dose aspirin or LMWH alone was suggested to be effective in the management of thrombophilia [86]. However, the ESHRE guideline does not suggest using antithrombotic prevention for females with hereditary thrombophilia and a history of recurrent miscarriage if it is not indicated for VTE prevention [4].

Many studies investigated different regimens of LMWH and/or low-dose aspirin therapy for women with thrombophilia-associated RPL [181,182,183,184,185,186,187]. Badawy et al. (2008) studied LMWH (enoxaparin 20 mg/day) in women with a history of three and more miscarriages [183] and recommended the therapy until 34 weeks of gestation. Clark et al. (2010) evaluated the effect of LMWH (enoxaparin 40 mg/day) and aspirin (75 mg/day) from the first trimester (before seven weeks’ gestation) until 36 weeks [184]. Martinelli et al. (2012) investigated the effect of LMWH (nadroparin 3800 IU/day) in women with RPL [185]. Another study compared the effect of three different anticoagulant regimens for the management of women with RPL during the first trimester of pregnancy: (1) aspirin 100 mg daily; (2) LMWH (enoxaparine 40 mg/day); (3) aspirin 100 mg and LMWH (enoxaparine 40 mg/day) [186]. The comparison of the three treatment options’ effect showed that “all three treatment regimens were significantly effective comparing live births against fetal losses” [186]. Thus, research data suggest a significant protective effect of anticoagulant therapy against recurrent miscarriages. Moreover, patients with APS treated with low-dose aspirin and LMWH during pregnancy should continue the therapy during the postpartum period (6–12 weeks) to ensure the reduction of the thrombotic events risk [187].

To improve live birth rates and prevent further miscarriage in patients with RPL associated with APS, all guidelines unanimously recommend combined therapy with low-dose aspirin and unfractionated heparin or LMWH [3,4,5,6,9,15,18,19,112,114]. However, only the DGGG/OEGGG/SGGG and ESHRE guidelines specify the initiation time for the anticoagulant therapy, which should begin with a positive pregnancy test [4,19]. The RCOG guideline does not suggest the use of corticosteroids nor intravenous immunoglobulin (IVIG) therapy for managing APS in RPL patients [3], as there are no facts supporting the live birth rate improvement compared with other treatment options. However, the recent meta-analysis of 54 RCTs comprising 4957 participants supports the efficacy of treatment with hydroxychloroquine, IVIG, and prednisone when prescribed in addition to the first-line therapy with low-dose aspirin and LMWH [114]. Based on a recent RCT [188], ESHRE suggested the administration of “repeated and high doses of IVIG very early in pregnancy” that could be beneficial for the improvement of live birth rates in women with four or more idiopathic pregnancy losses [5].

Additional large-scale, high-quality RCTs are suggested focusing on RPL patients to investigate and confirm the efficacy of IVIG therapy for recurrent miscarriage prevention [4,108].

For women with RPL associated with the MTHFR gene mutation in and/or hyperhomocysteinemia, treatment with folic acid is recommended to prevent further miscarriages [4,189].

#### 3.6.6. Treatment of Recurrent Pregnancy Loss Related to Immune Factors

The results of existing clinical studies and meta-analyses on the administration of corticoids to patients with a history of pregnancy loss for lowering the risk of RPL are inconsistent [19,112,167,190].

According to the RCOG and ESHRE guidelines, corticoids and IVIG are not recommended as treatments for women with recurrent miscarriage with selected immunological biomarkers as these medications do not improve the live birth rate of women with a history of RPL [4,5,112]. Moreover, this type of treatment is expensive of potential allergic reactions and is suggested for patients with pre-existing autoimmune diseases, which require corticosteroid hormone therapy during pregnancy [19,112].

Studies on IVIG during pregnancy for the purpose of the reduction of NK cell activation in peripheral blood were performed in women with idiopathic RPL without specific immunological factors defined as responsible for pregnancy loss [19,112,191]. Thus, currently, no evidence to support the administration of IVIG.

Intralipid treatment with 20% sterile fat emulsion containing soybean oil, phospholipids, glycerin, and water is recommended for immune response modulation [112]. It potentially could be used in patients with RPL associated with immune factors. However, none of the available guidelines suggest using it for women with RPL [4,6,88,166]. Only the DGGG/OEGGG/SGGG guideline considers intralipid infusion as a therapeutic option for women with recurrent miscarriage within clinical studies, not routinely [19].

#### 3.6.7. Vitamin D Supplementation

Identification of the exact causes of inappropriate vitamin D activity, stemming from a deficiency in vitamin D levels, inappropriate signaling through VDR, or heightened vitamin D-binding protein levels, is instrumental in instituting effective replacement therapy. Some researchers investigated in RCTs and case-control studies the effect of vitamin D on the risk of RPL [192,193,194,195], however, they did not find a significant difference in the rates of RPL between patients who were treated with vitamin D and controls [192,193]. Moreover, a recently published meta-analysis by Tamblyn et al. (2022) reports insufficient evidence to suggest vitamin D treatment for the reduction of the RPL risk in women with a history of pregnancy loss [138]. Thus, the relevance of preconception therapy with vitamin D for RPL prevention remains unclear [137]. Additional studies are required to develop an evidence-based strategy for vitamin D supplementation for preconception counseling and during pregnancy [138,151].

### 3.7. Psychological Effect of Recurrent Miscarriage

Family is the basis and the bedrock of society. It is especially strong in orthodox religious societies, which follow the traditional beliefs and roles of family members [14,196,197]. The role and the place of a woman in such cultures depend on her ability to conceive and give birth to healthy offspring [12,196,198,199]. Repeated loss of a planned and desirable pregnancy is a distressing life event for a female [200]. Pregnancy loss leads to a significant emotional and subsequent psychological impact on women and their partners [4,15,200], including grief, filling of guilt, fear of the future, relationship/communication conflict, marital distress, and poor personal adaptation [1,201,202]. The severity of psychological consequences of RPL is associated with maternal age, gestational age at pregnancy loss, and a number of previous miscarriages [201]. If not properly managed, these emotional experiences may lead to the development of a variety of psychological conditions, such as stress, depression, anxiety, and severe psychiatric morbidity [1,200,202]. However, the impact of psychological factors on women’s physical health and reproductive morbidity and its impact on the incidence of further pregnancy losses is underestimated [199]. Moreover, some studies report the role of psychological conditions as a possible primary etiology for recurrent miscarriage [203,204].

The available results of research on the psychological morbidity of women with RPL reported high levels of stress, depression, and anxiety [200,204,205,206,207,208], suggesting these symptoms contribute to the increased risk of subsequent pregnancy loss [200,206].

All available international guidelines on RPL management highlight the importance and value of psychological support to couples, which could help to decrease the risk of further pregnancy losses [3,4,5,6,19]. However, a limited number of publications are available on the effect of psychological interventions in improving pregnancy outcomes in women/couples suffering from RPL [4,209,210]. Most of the available investigations are self-reporting survey-based studies, which do not include male partners [211]. It affects a deep understanding of the perspectives of couples experiencing repeated miscarriages.

Available systematic reviews of cohort studies and RCTs suggest that psychological support and interventions “may improve pregnant women’s psychological well-being after miscarriage” [209,212], which could reduce adverse pregnancy outcomes in subsequent gestation. Recently published RCT by Jensen et al. (2021) reported a “tailored meditation and mindfulness intervention” for women with RPL and proved that a 7-week daily home-based “meditation and mindfulness programme combined with group sessions reduced perceived stress significantly more than a standard supportive care programme” [210].

However, the number of such reports and the availability of psychological support services are limited, especially in low-income settings. To identify the effective approach and methods for the prediction and prevention of psychological morbidity associated with RPL, further research, and screening for mental health issues after RPL is of paramount importance [10,200]. Future research studies and healthcare professionals should consider the psychosocial needs of couples suffering from RPL while creating a care and management plan for these couples [4,210].

## 4. Conclusions

Recurrent miscarriage is a traumatic life event that affects women’s physical and psychological health and social well-being. Different international guidelines on RPL management are approved and implemented into clinical practice. However, these guidelines follow different definitions of RPL, thus making the estimation of recurrent miscarriage epidemiology inaccurate. Moreover, based on the variations in definitions, these guidelines offer a specific management plan either after two or three cases of pregnancy loss, which makes the general approach to the condition inconsistent. In addition, there is an increasing number of work-up and therapeutic options offered to women with RPL. These practice variations should be solved by the implementation of evidence-based recommendations. Grounded on the up-to-date guidelines, the following risk factors should be investigated in patients with RPL: chromosomal abnormalities, congenital and acquired uterine pathologies, endocrine disorders, thrombophilia, and autoimmune diseases. The management offered to patients should be based on the diagnostic findings and based on the existing guidelines’ recommendations.

The international guidelines require regular updates as new insights on the risk factors and novel management methods are being developed. Studies to identify etiology and risk factors for RPL, especially idiopathic, should be continued. Knowledge of specific genes contributing to RPL could help in understanding the biological pathways of the condition and, thus, shed light on the proper management approach. Available live birth prediction models could assist in the management of couples with RPL. However, more evidence is required to clarify whether treatments with corticosteroids, IVIG, and vitamin D are justified for patients with RPL due to these factors’ abnormalities.

## Figures and Tables

**Table 1:** Comparison of international guidelines’ definitions/diagnostic criteria for recurrent pregnancy loss.

Criteria	Guidelines (Year of Publication)				
	WHO (1977)	RCOG (2011)	ASRM (2012/2013)	ESHRE (2017/2022)	DGGG/ÖGGG/SGGG (2018)
**Number of RPLs (n)**	3	3	2	2	3
**Sequence of loss**	Not specified	Consecutive	Consecutive	Consecutive and non-consecutive	Consecutive and non-consecutive
**Type of pregnancy**	All pregnancy losses, types are not specified	All pregnancy losses, types are not specified	Clinical pregnancies	Non-visualized pregnancy losses (biochemical pregnancy losses and/or resolved and treated pregnancies of unknown location), clinical pregnancies	All pregnancy losses, types are not specified
**Evidence of loss**	Not specified	Not specified	US or histopathologic examination	Serum or urine β–hCG; US	Not specified
**Gestational age at pregnancy loss (weeks)**	20 (22)	24	The first trimester of pregnancy, gestational weeks not specified	24	24
**Fetal weight (grams)**	<500	Not specified	Not specified	Not specified	<500

ASRM—American Society of Reproductive Medicine; DGGG/ÖGGG/SGGG—German Society of Gynecology and Obstetrics/Austrian Society of Gynecology and Obstetrics/Swiss Society of Gynecology and Obstetrics; ESHRE—European Society of Human Reproduction and Embryology; RCOG—Royal College of Obstetricians and Gynecologists; RPL—recurrent pregnancy loss; US—ultrasonography; WHO—World Health Organization; β-hCG—β human chorionic gonadotropin.

## Data Availability

Due to the nature of this study, narrative review, no data are available for sharing.

## Supplementary Materials

The following supporting information can be downloaded at: https://www.mdpi.com/article/10.3390/jcm12124074/s1, Table S1: Search strategy.
