# Supplementary material for: Recurrent Pregnancy Loss Etiology, Risk Factors, Diagnosis, and Management. Fresh Look into a Full Box
Source: J Clin Med. 2023 Jun 15;12(12):4074. doi: 10.3390/jcm12124074 (PMC10298962; doi:10.3390/jcm12124074)
Supplement: Supplementary file 1 [file jcm-12-04074-s001.zip › jcm-2397654-supplementary.pdf]

Table S1. Search strategy.

Keyword	MeSH Unique ID
Spontaneous pregnancy loss	-
Early pregnancy loss	-
Spontaneous abortion	D000022
Recurrent pregnancy loss	-
Recurrent miscarriage	-
Recurrent early pregnancy loss	-
Habitual abortion	D000026
Missed abortion	D000030
Incomplete abortion	D000027
Embryo loss	D020964
Pregnancy complications	D011248
Progesterone	D011374
Luteal phase deficiency	-
Luteal phase	D008183
Factor V Leiden	C095381
MTHFR deficiency	C565512
Methylenetetrahydrofolate reductase deficiency	C537357
Homocysteine	D006710
Hyperhomocysteinemia	D020138
Antiphospholipid syndrome	D016736
Uterine diseases	D014591
Vitamin D deficiency	D014808
Psychological stress	D013315
Anxiety	-
Anxiousness	D001007
Depression	-
Depressive symptom	D003863
